# Trueness and Precision of Two Intraoral Scanners: A Comparative In Vitro Study

**DOI:** 10.1155/2019/1289570

**Published:** 2019-10-21

**Authors:** Raul Nicolae Rotar, Anca Jivanescu, Codruta Ille, Angela Codruta Podariu, Daniela Elisabeta Jumanca, Ana-Maria Matichescu, Octavia Balean, Laura Cristina Rusu

**Affiliations:** ^1^Department of Prosthodontics, University of Medicine and Pharmacy “Victor Babes”, Timisoara, B-dul Revolutiei 1989, No 9, 300580, Romania; ^2^Department of Preventive Dentistry, Community and Oral Health, University of Medicine and Pharmacy “Victor Babes”, Timisoara, Splaiul Tudor Vladimirescu, nr.14 A, 300174, Romania; ^3^Department of Oral Pathology, University of Medicine and Pharmacy “Victor Babes”, Timisoara, Splaiul Tudor Vladimirescu, nr.14 A, 300174, Romania

## Abstract

The aim of this study was to evaluate the accuracy of two intraoral scanners used in the dental office. A molar fixed in a typodont was prepared for a ceramic onlay. The preparation was scanned using a high-resolution scanner (reference scanner) and saved as stereolithography (STL) format. The prepared resin molar was scanned again using the intraoral scanners, and all the scans were saved as well in STL format. All STL files were compared using metrology software (Geomagic Control X). Overlapping the meshes allowed the assessment of the scans in terms of trueness and precision. Based on the results of this study, the differences of trueness and precision between the intraoral scanners were minimal.

## 1. Introduction

Digital impressions are getting more and more importance in the dental office, leading to an increase in the number of intraoral scanners available on the market [1–5], and as a result, many clinicians may have second thoughts when choosing the most suitable intraoral system for their work [2, 6].

The main advantages that these systems provide over the conventional impression are the comfort for the patient, time efficiency, and also the reduced costs [7, 8]. Also, the possibility of immediate control of the impression and basically “indestructible” 3D models that can be stored indefinitely add up to the scale in favor of digital impression procedures [9–11].

The way a scanner works is by measuring the reflection times of a surface and based on an algorithm it “attaches” the images that it records. Even if the digital impression procedure is not very complicated, the working algorithm is complex [1]. The scanner's software generates point clouds and meshes that reconstruct the scanned surface using a powerful processing software that allows for high-quality 3D models [12, 13].

A number of studies have shown that intraoral scanners are a reliable way of recording tooth preparations whether they are single crowns, inlays, onlays, implants [14–16], or fixed partial dentures [17–19].

When comparing digital impression accuracy, there are two aspects that are taken into consideration: trueness and precision. These variables are independent and do not reflect the same thing [13, 14]. Trueness shows how similar is a measurement to the value of the measured quantity. On the other hand, precision shows how much similar are repeated measurements, in other words the reproducibility of the impression [13–15]. As a result, the ideal intraoral scanner should have high trueness and also high precision.

The most common way of measuring the accuracy or either conventional or digital impressions is by comparing a reference scan, usually obtained by scanning a physical model with a desktop or an industrial scanner, and the resulting STL file is then compared with the test scan groups [20–24].

Due to the fact that there is no standardized method of scanning and the acquisition techniques for the IOS differ from one system to another, the analysis of the resulting meshes may prove difficult [21, 25].

A precise fit is extremely important when referring to long-lasting dental restorations. As a result, the impression process becomes a key step in determining the success of a treatment. A precise impression allows for a clear identification of the finish line which translates into a suitable emergence profile [26–29].

The aim of this study was to compare the accuracy (trueness and precision) of two intraoral scanners on an onlay preparation and to assess if there are any major discrepancies between the qualities of the final digital impressions.

## 2. Material and Methods

Two intraoral scanners Planmeca PlanScan (E4D Technologies, LLC, Richardson, TX, USA) and CEREC Omnicam (Sirona, Bensheim, Germany) and a high-resolution desktop scanner D700 (3Shape, Copenhagen, Denmark) were used in this study.

The Planmeca PlanScan works under the principle of optical coherence tomography and confocal microscopy. It is a powder-free scanner with a blue light real-time laser video streaming technology. It has tips of various dimensions with built-in heated mirrors. Planmeca PlanScan is an open system, since it allows conversion of the acquired proprietary files into STL files, readable by all CAD systems. It can be easily connected to a laptop via a USB port and has a proprietary milling machine available for the fabrication of full in-office digital restorations such as inlays, onlays, crowns, bridges, and veneers.

CEREC Omnicam is a structured light scanner that uses a white LED, and it works under the principle of optical triangulation and confocal microscopy. It is fast, it does not require powder, and it offers true color information. The tip is not too big; therefore, it is easier to scan the posterior areas. The digital workflow can take place directly at the chairside, using the proprietary CAD software, or via the cloud-based platform. CEREC Omnicam is also an open system allowing transformation of proprietary files into STL files, usable from any CAD system. The CAD/CAM system of Sirona allows the design and milling of prosthetic restorations and frameworks (inlays, onlays, veneers, crowns, bridges, and bars).

D700 is a desktop scanner that uses two cameras with reduced angle that allows the scanning of deep preparations and undercuts. It has a high accuracy (<20 microns) and is material color independent.

**Figure 1:**
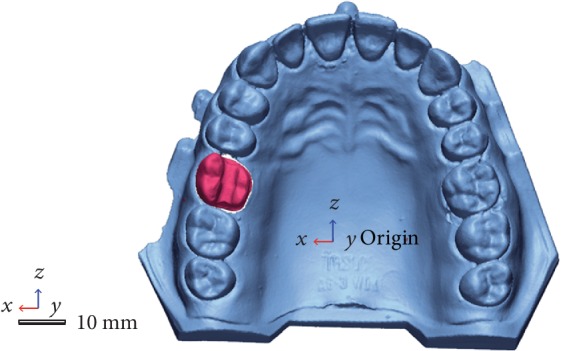
Reference model.

A standard resin upper first molar was prepared for a ceramic onlay. Next, the model was digitized using a desktop scanner (D700, 3Shape) in order to obtain a reference model. First, the prepared tooth was removed from the typodont and scanned individually followed by another scan with the adjacent teeth that later served in the alignment process. The 3-axis motion system facilitated easy object placement allowing the object to be tilted, rotated, and translated so as to be scanned from any viewpoint, making 3-axis the optimal number of axis for a scanning volume corresponding to a dental model. In the final processing step, the point cloud obtained from all views was converted into a 3D surface of fine triangles and the resulting data saved as a STL file (Figure 1).

**Figure 2:**
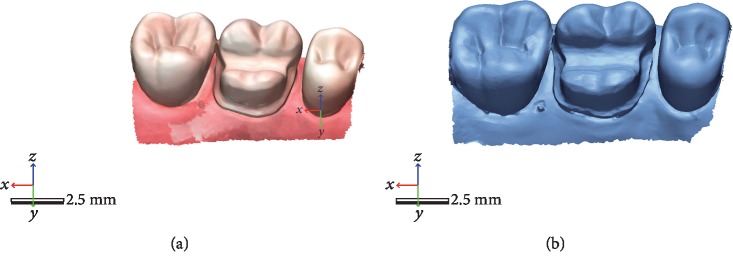
Intraoral scans with PlanScan (b) and Omnicam (a).

The same prepared molar was scanned ten times using two high-end intraoral scanners. The first five scans were taken with the scanner from Planmeca PlanScan and the rest up to ten with the Omnicam from CEREC. A specific scanning pattern was followed for all the scans starting from the mesial part of the occlusal surface of the preparation and then transitioning to the palatal surface followed by the distal part of the occlusal surface and in the end the transition to the buccal side of the prepared tooth, all in a continuous motion. All files were saved in STL format as well and used later on for a comparison in terms of trueness and precision (Figure 2).

**Figure 3:**
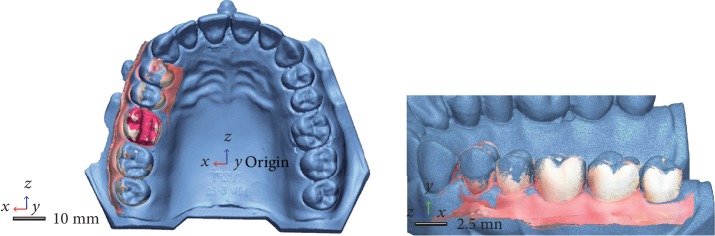
Alignment process of the meshes.

Trueness values were obtained by superimposing the STL files from the test groups with the STL file from the reference scan. Overlapping the STL files within each group generated the precision values. Two random scans from each intraoral system were chosen and compared with all the other meshes from within each group. All scanning data and computations were performed using metrology software (Geomagic Control X). Using reverse engineering, the STL files were uploaded into the program and the models were trimmed, and only the prepared tooth data was analyzed. The STL file from the desktop scanner was set as the reference. The 3D models from the intraoral scanners were superimposed in the beginning using a rough “initial alignment” followed by a “best fit algorithm” that determined the final overlapping of the meshes (Figure 3). The resulting color map of the analyzed meshes was set between ±50 *μ*m. The distances between different planes were color-coded, and the overall color map was generated based on these deviations.

**Figure 4:**
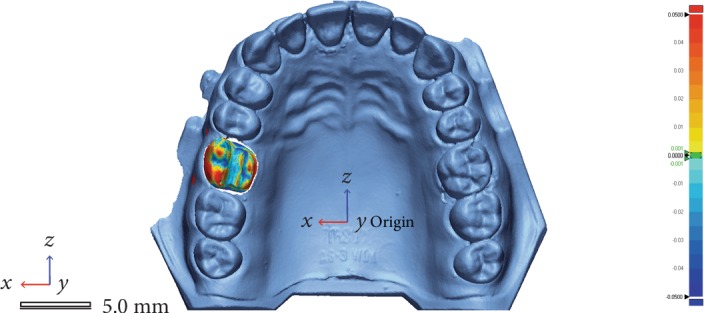
Color map of the deviation on the interest area.

For each set of scans, the mean and standard deviation values were calculated. The blue color indicated the inward displacement, and the red color showed the outward position of the mesh compared to the reference while the green color showed the absence of change (Figure 4).

Statistical analysis was preformed using the Kolmogorov-Smirnov test to assess data distribution.

Overall trueness and precision of the scanners were analyzed and compared, and the statistical significance was calculated using the paired *t*-test.

## 3. Results

The trueness and precision values of the two intraoral scanners for the onlay preparation are presented in Tables 1 and 2, respectively.

The mean trueness value of 48.6 ± 4.39 *μ*m showed that the PlanScan scans had the best overall results. Regarding the precision of the two intraoral scanners, PlanScan also showed better results with a mean value of 24.86 ± 2.91 *μ*m.

The *p* values for both trueness and precision were >0.05, indicating that there was no difference between the scanners.

**Figure 5:**
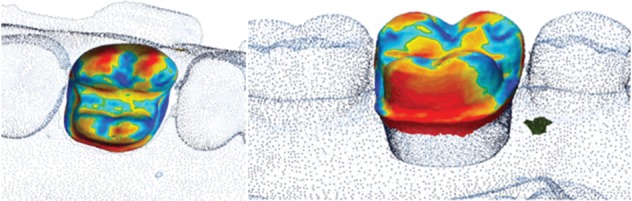
Color map of the PlanScan trueness deviation values around the interest area.

**Figure 6:**
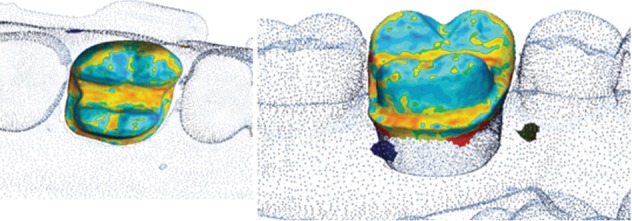
Color map of the Omnicam trueness deviation values around the interest area.

**Figure 7:**
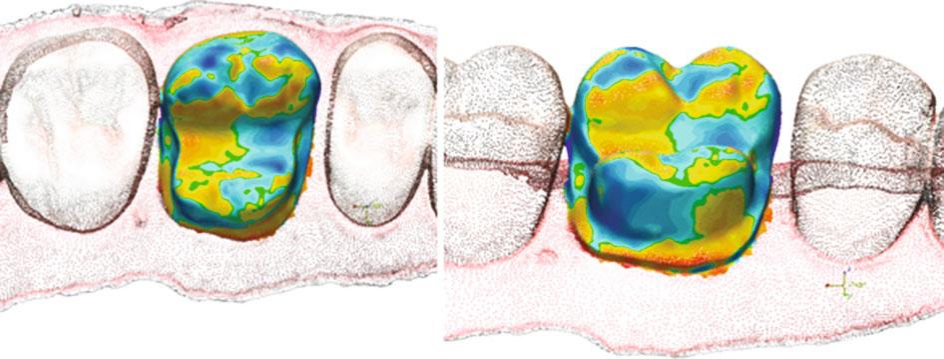
Color map of the PlanScan precision deviation values around the interest area.

**Figure 8:**
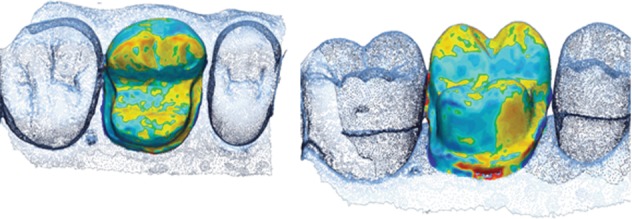
Color map of the Omnicam precision deviation values around the interest area.

The single best results for trueness and precision (visual color map representation) obtained with each device are presented in Figures 5–8.

## 4. Discussions

Clinical practice in dentistry is changing at an incredible pace due to the developments that take place in the software (computer assisted design) and hardware (milling machines and scanning tips) fields [1, 3, 5]. Optical impressions enhance the workflow in the dental office that leads to more predictable results, allowing for real-time adjustments of the impressions and when needed corrections of the prepared tooth areas [3, 9].

With so many intraoral scanners available on the market, little is known about the accuracy (trueness and precision) of these devices [10, 12]. A number of studies have shown that even if intraoral scanners are a reliable way of recording tooth preparations, it is not clear if they can completely replace the conventional impression in all treatment plans [15–17].

Nedelcu et al. assessed the accuracy of four intraoral scanners and concluded that these devices should be used only in particular scenarios that include smaller prosthetic treatments [21].

Similar conclusions were drawn by Schaefer et al. who measured the marginal fit of partial ceramic crowns and showed that even if the marginal gap distances were acceptable, there were important differences between the scanning systems [30].

Andriessen et al. also measured and compared the accuracy of three intraoral scanners for 3 implants on an edentulous ridge. The conclusion of the study was that the errors are directly proportional with the size of the scanned surface [15].

Our study has a number of limitations. Being an *in vitro* study, aspects that can influence the final accuracy of the digital impression such as humidity, saliva, blood, patient's movements, or the space for the scanning tip were not taken into consideration.

As a result, the observations of this study may be subject to change as the developing companies are investing more and more for the improvement of the data acquisition of these intraoral scanning systems.

## 5. Conclusions

This study compared the trueness and precision of two intraoral scanners in the scenario of an onlay on a complete dentate arch. The accuracy deviations of the analyzed scanners were consistent and with no major differences between them. Even if there were some deviations in visual inspection of the meshes, there was no statistical significance between the two intraoral scanners. More *in vivo* and *in vitro* studies are necessary for a clear validation of these results.

## Figures and Tables

**Table 1:** Trueness values (*μ*m) of the intraoral scanners (*p* value = 0.2).

	M1	M2	M3	M4	M5	Mean ± SD
Planmeca PlanScan	43 *μ*m	53 *μ*m	46 *μ*m	53 *μ*m	48 *μ*m	48.6 ± 4.39 *μ*m
CEREC Omnicam	54 *μ*m	53 *μ*m	46 *μ*m	50 *μ*m	62 *μ*m	53 ± 5.91 *μ*m

**Table 2:** Precision values (*μ*m) of the intraoral scanners (*p* value = 0.08).

	M1	M2	M3	M4	M5	M6	M7	Mean ± SD
Planmeca PlanScan	28 *μ*m	25 *μ*m	21 *μ*m	28 *μ*m	22 *μ*m	27 *μ*m	23 *μ*m	24.86 ± 2.91 *μ*m
CEREC Omnicam	31 *μ*m	31 *μ*m	55 *μ*m	21 *μ*m	39 *μ*m	28 *μ*m	44 *μ*m	35.57 ± 11.34 *μ*m

## Data Availability

All data is available upon request.
